# New insights into the life cycle, host cell tropism, and infection amplification of *Leishmania* spp.

**DOI:** 10.1128/iai.00123-25

**Published:** 2025-06-13

**Authors:** Anna Luiza Silva-Moreira, Artur Metzker Serravite, Laura Valéria Rios-Barros, Juliana Perrone Bezerra de Menezes, Maria Fátima Horta, Thiago Castro-Gomes

**Affiliations:** 1 Departamento de Parasitologia, Universidade Federal de Minas Gerais 28114, Belo Horizonte, Minas Gerais, Brazil; 2 Instituto Gonçalo Moniz, Fundação Oswaldo Cruz, Bahia, Salvador, Brazil; 3 Departamento de Bioquímica e Imunologia, Instituto de Ciências Biológicas, Universidade Federal de Minas Gerais, Belo Horizonte, Minas Gerais, Brazil; University of California Merced, Merced, California, USA

**Keywords:** *Leishmania *spp, leishmaniasis, host cells, infection amplification, life cycle

## Abstract

The genus *Leishmania* comprises several species of digenetic protozoan parasites that cause a spectrum of diseases known as leishmaniases, which are transmitted to humans and other mammals through the bite of hematophagous female sand flies. *Leishmania* spp. and their invertebrate vectors are widely distributed across the globe, putting more than a billion people at risk. Once inside mammalian hosts, these intracellular parasites reside within parasitophorous vacuoles of host cells. Although macrophages are the primary infected cells in lesions, *Leishmania* can also infect other cell types, whose roles in maintaining the parasite’s life cycle and contributing to pathogenesis remain unclear. Similarly, the processes governing parasite dissemination from the initial infection site in the skin to internal organs, as well as the mechanisms driving the infection of new cells, are still under investigation. In this review, we underscore some existing gaps in *Leishmania*’s life cycle, discussing i) the various cell types that serve as host cells for the parasite and their potential roles in the disease, ii) the mechanisms that might contribute to infection amplification, iii) the strategies possibly involved in dissemination and visceralization, iv) the mechanisms driving the generation of super-infective vectors, and v) the occurrence of a mating stage in the cycle. Altogether, these aspects may reshape our perspective on the basic biology of *Leishmania*, deepening our understanding of the host-parasite relationship and hopefully opening avenues toward a better understanding of the disease.

## LEISHMANIASES AND *LEISHMANIA* SPP

Leishmaniases comprise a group of neglected diseases caused by pathogenic protozoa of the genus *Leishmania*, transmitted to vertebrate hosts through the bite of female sand flies infected with the parasite (1). They occur in all continents across the globe, except for Oceania, and are particularly endemic to South America, South Asia, the Middle East, the Mediterranean, North Africa, and Sub-Saharan Africa (2). Currently, more than one billion people live in areas endemic for leishmaniasis and are at risk of infection. It is estimated that 50,000 to 90,000 new cases of visceral leishmaniasis (VL) and 600,000 to 1 million new cases of cutaneous leishmaniasis (CL) occur annually (1). There are around 54 species of *Leishmania* described, of which at least 21 are pathogenic to humans (3) and which can be transmitted by more than 100 species of sand fly vectors (4). The two main forms, CL and VL, are determined by the species of the parasite involved. There are dozens of species that cause CL, the more prevalent being *L. braziliensis, L. amazonensis*, *L. mexicana*, *L. guyanensis*, and *L. panamensis*, (in the New World) and *L. major*, *L. tropica,* and *L. aethiopica* (in the Old World) (3). The visceral form, also known as kala-azar, which affects internal organs, is primarily caused by *L. infantum* and *L. donovani*. While these species are typically associated with VL, species usually causing cutaneous forms may progress to affect internal organs, in immunocompromised individuals, resulting in visceralization (1). In VL, the presence of parasites is observed in cells of internal organs such as liver, spleen, and bone marrow, and the disease is characterized by intermittent fever episodes, substantial weight loss, swelling of the spleen and liver, and pancytopenia with severe anemia. If untreated, the disease can lead to death, with a mortality rate of 95% (1). On the other hand, CL manifests as skin lesions (nodular or ulcerated) with different clinical manifestations, depending on both the species of parasite and the immune status of the host. In mucocutaneous leishmaniasis (MCL), the disease is characterized by lesions that can partially or completely destroy the mucus membranes of the nose, mouth, throat, and surrounding tissues (1). Another severe form of the cutaneous manifestation of the disease is disseminated leishmaniasis (DL), commonly associated with immunosuppressed patients, in which parasites are spread throughout the skin, causing the appearance of numerous non-ulcerated nodular lesions. Although there are available drugs to combat the parasite in humans, such as amphotericin B, meglumine antimoniate, and miltefosine, treating leishmaniases is still a public health challenge: the therapeutic regimen is too long, and some drugs can be highly toxic or frequently cause impactful side effects, making it difficult for patients to adhere to treatment. Recent therapeutic clinical trials combining two leishmanicidal drugs have not been encouraging, particularly for the strains circulating in Latin America (5). Furthermore, relapses of the disease occur after completion of treatment and may even be underestimated (6).

In the epidemiological chain of leishmaniases, several mammalian species (including humans, dogs, and wild mammals) serve as reservoirs, where the parasites reproduce as amastigotes and infect sand flies. Once contaminated, and after re-transformation to promastigotes and development of infective metacyclic forms, sand flies can transmit the infective forms to humans and other mammals through subsequent blood meals. Understanding the transmission chain and the diversity of reservoirs and vectors of *Leishmania* spp. is, therefore, fundamental to developing measures of containment and epidemiological surveillance. Although the domestic dog is the most important reservoir for VL transmission in urban areas, there has been an increasing number of reports of the disease in other animals, including non-human primates (South America) (7), wild rabbits (Mediterranean) (8), cattle (Middle East) (9), wild rodents (South America and Mediterranean) (10, 11), and bats (South America) (12).

The urbanization of wild habitats can influence the transmission of leishmaniasis by bringing humans, reservoirs, and vectors closer to new reservoirs and vectors. Semi-urban environments can provide suitable habitats for species that function as reservoirs and for sand fly vectors, creating favorable conditions for disease transmission (13). Furthermore, global warming, driven by greenhouse gas emissions, significantly impacts vector-borne diseases, like leishmaniasis. The increase in average temperatures can directly prolong the survival of vectors and, consequently, the rates of human exposure to infectious bites. Climate can also affect the abundance of vectors or their natural predators, interspecies competition, and thus, the dynamics of disease transmission as well as its geographic distribution and the resurgence of diseases through several pathways, including direct effects on pathogens, vectors, and hosts, and alter the entire ecosystem in which they inhabit (14). As an example, environmental and climatic changes have enabled sand flies to expand northward beyond their traditional European geographic distribution, impacting areas once considered free from leishmaniasis, including northern Italy, Germany, and Belgium (15). Additionally, human migratory flows driven by political conflicts can also play a significant role in epidemiological chains and have been linked with the increased incidence of CL in many countries (16–19).

## LEISHMANIA’S LIFE CYCLE: HOST CELLS, KNOWLEDGE GAPS, AND NEW FINDINGS

**Fig 1:**
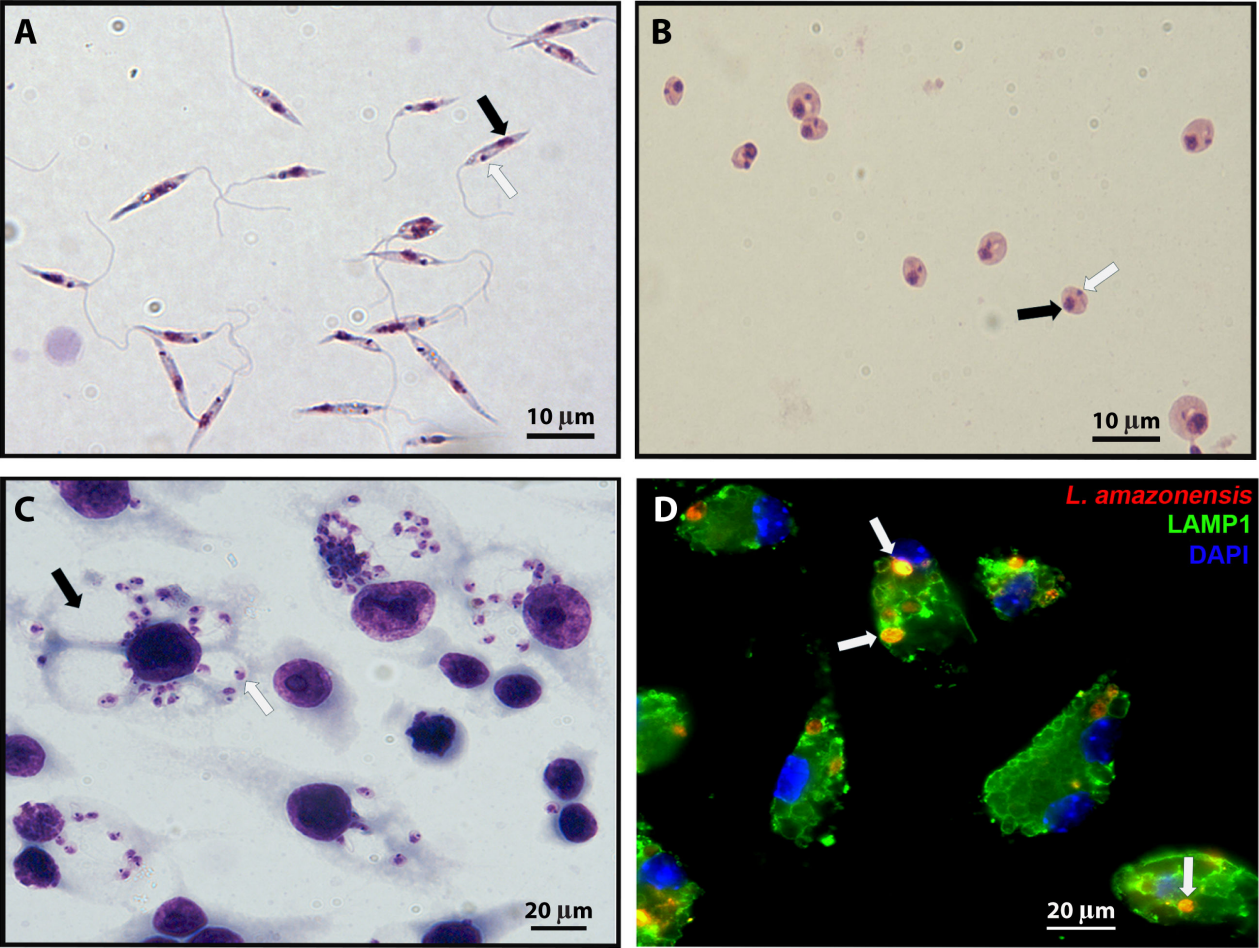
*Leishmania* infective forms outside host cells and within intracellular vacuoles. (A) Promastigote and (**B**) amastigote forms of *L. amazonensis* axenically cultivated, fixed, and stained by the Leishman staining technique. Black arrows show parasite nuclei, and white arrows show their kinetoplasts. (**C and D**) Macrophage-like cells (RAW cells) infected by *L. amazonensis* (10 parasites/cell). (C) Infected RAW cells were fixed after 24 h infection with promastigotes and stained by May-Grünwald-Giemsa. Light microscopy analysis showing intracellular amastigotes (white arrows) harboring typical vacuoles (black arrows). (D) RAW cells infected by *L. amazonensis* expressing red fluorescent protein were fixed after 24 h infection with promastigotes and labeled with LAMP1 antibodies (green) to show lysosomes and DAPI to visualize host cell nuclei (blue). Fluorescence microscopy analysis shows the intracellular amastigote forms colocalized with the lysosomal marker.

**Fig 2:**
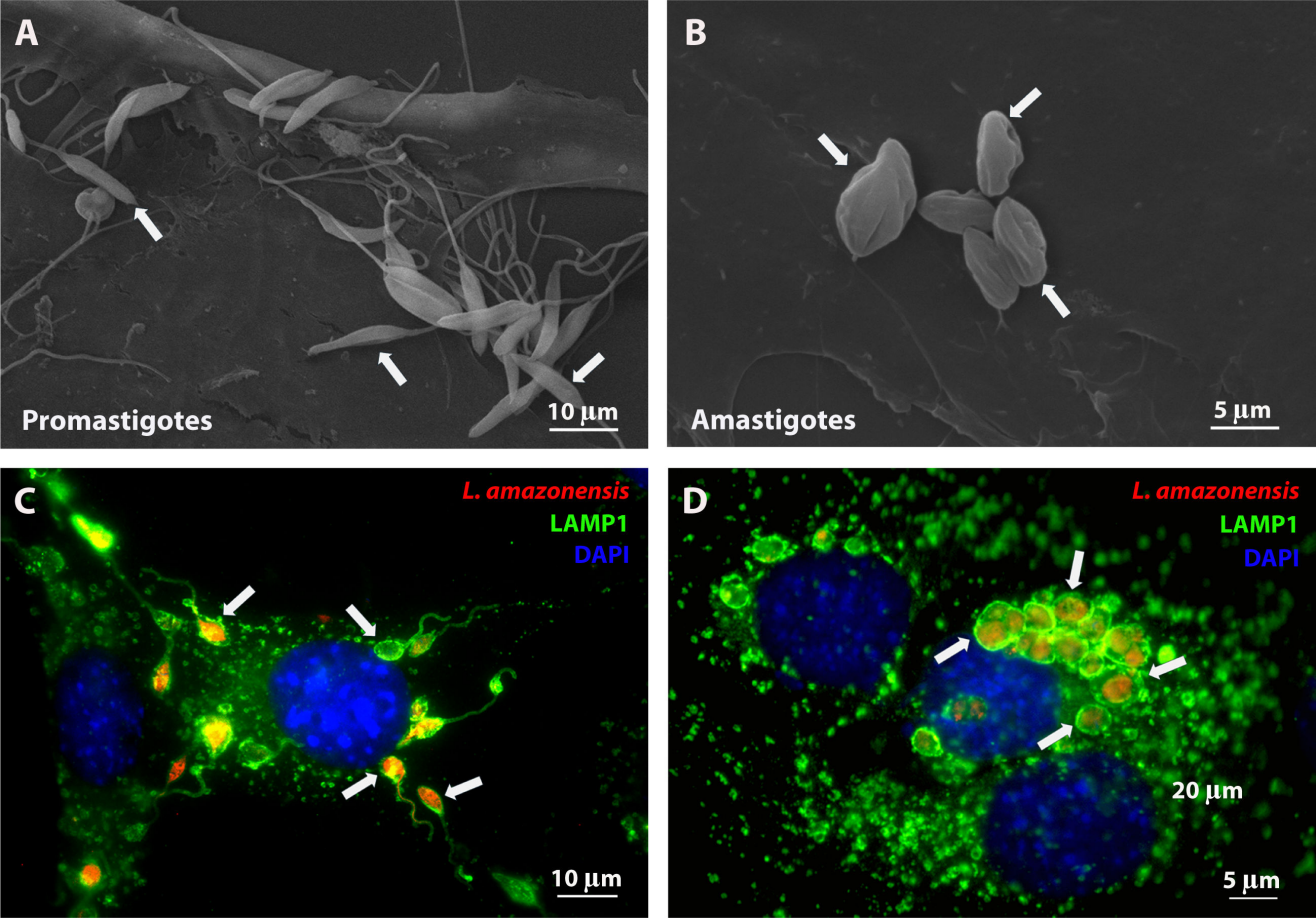
*Leishmania* infective forms also invade non-phagocytic cells. (A) Promastigote and (**B**) amastigote forms of *L. amazonensis* axenically cultivated interacting with mouse embryonic fibroblasts (MEFs) during cell invasion and analyzed by scanning electron microscopy (promastigotes = 25 parasites/cell and amastigotes = 10 parasites/cell). White arrows show some parasites on the membrane of the fibroblasts. (**C and D**)Promastigotes and amastigotes of *L. amazonensis* expressing red fluorescent protein 1 h after incubation with MEFs. Cells were fixed and labeled with LAMP1 antibodies (green) to show lysosomes and DAPI to visualize host cell nuclei (blue). Fluorescence microscopy analysis reveals that the internalized parasites are located in PVs, whose membranes are rich in Lamp1, a lysosomal marker, indicating their extensive interactions with late endocytic pathway organelles. White arrows show some parasites inside PVs.

**Fig 3:**
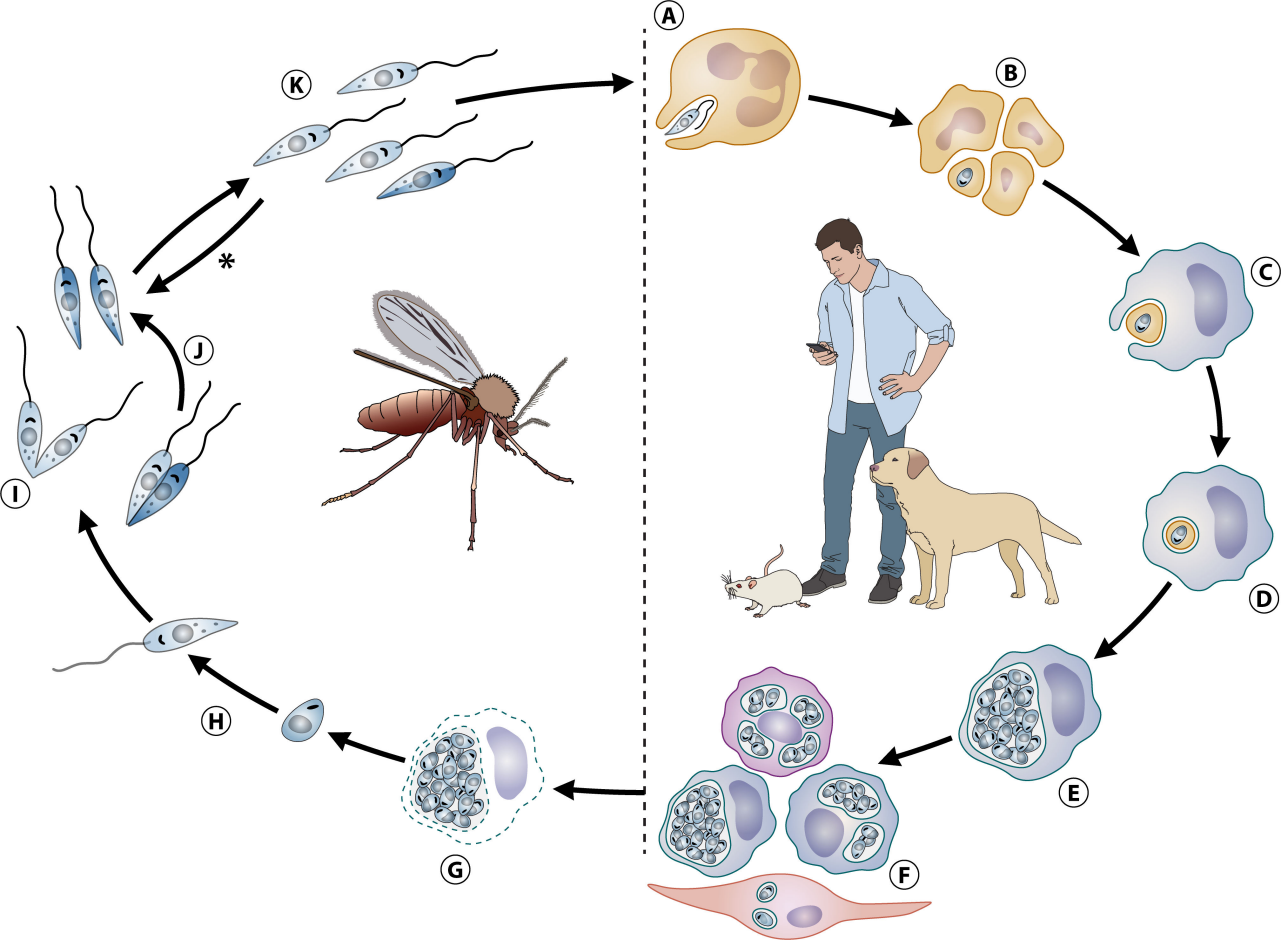
Life cycle of *Leishmania* spp. (A) The female infected sand fly regurgitates infectant metacyclic promastigotes into the dermis of a mammalian host during a blood meal, infecting neutrophils attracted to the site of the bite. (B) The parasites transform into intracellular amastigotes before neutrophils undergo apoptotic death. (C) Apoptotic bodies of dead infected neutrophils are captured by macrophages, (D) which become infected. (E) Amastigotes multiply by binary fission in intracellular. acidic vacuoles rich in lysosomal markers, increasing the number of parasites per infected cells through time. (F) By mechanisms that remain poorly understood, infection propagates, and the number of infected cells also increases, leading to infection amplification. Macrophages are the main cell found infected, although other cell types have also been characterized as harboring intracellular amastigotes in infected tissues. Infection of the invertebrate host (vector): (G) The female sand fly becomes infected after ingesting infected cells containing intravacuolar amastigotes during a blood meal on an infected host (commonly a parasite reservoir, such as the domestic dog or wild mammals). (H) In the digestive tract of the vector, amastigotes are released and transform into flagellated promastigotes, which (I) multiply by binary fission. (J) During this step of their life cycle, it has been demonstrated that a sexual-like cycle with hybridization of fused mating parasites also occurs. (K) Inside the sand fly digestive tract, the non-infective procyclic promastigotes undergo a sequence of biochemical and morphological changes until highly infective metacyclic promastigotes are generated, which are inoculated into the dermis of a mammalian host in a second blood meal. In the past, it was believed that the metacyclic forms of the parasite were terminally differentiated, but recent findings demonstrated that upon a second blood meal by the vector, the remaining metacyclic promastigotes can (*) de-differentiate into proliferative procyclic promastigotes, considerably increasing the number of parasites per vector and their infectiveness.

Female sand flies of the genera *Phlebotomus* (in the old world) and *Lutzomyia* (in the new world) are considered the only invertebrate vector hosts of *Leishmania* spp., in which their development as promastigote forms (Fig. 1A and 2A) takes place. During blood meals carried out by infected sand flies, promastigotes are transmitted to mammalian hosts (Fig. 3A). Although there are still gaps in our understanding of the parasite life cycle within mammalian hosts, particularly regarding the host cells (Fig. 3B and C and discussed below), it is generally correct to state that the parasites are ultimately found as amastigotes (Fig. 1B and 2B) primarily within macrophages at infection sites on the skin or parasitized organs. These amastigotes reside in typical vacuoles, the parasitophorous vacuoles (PV) (Fig. 1C, black arrow, Fig. 3D through F), which are acidic and enriched in lysosomal markers (Fig. 1D, white arrows).

*Leishmania* spp. are known for their ability to infect phagocytic cells of the immune system (i.e., neutrophils, dendritic cells (DC), and macrophages). Macrophages play a crucial dual role in Leishmania infection: they host the parasites, allowing their replication and propagation, while also being responsible for their elimination, depending on the host’s immune response. Therefore, they have been the most studied host cell both *in vitro* and *in vivo*, in natural or experimental infections with different species of *Leishmania*. However, it has been shown that the onset of the infection relies first on neutrophils, which are quickly attracted to the insect bite site. Below, we comment on the different cell types, phagocytic or not, reported as infected by *Leishmania* spp., both at the initial and at the amplification phase of infection by promastigotes or amastigotes, respectively.

## NEUTROPHILS AND MACROPHAGES—THE ONSET OF INFECTION

Although macrophages are capable of actively capturing the promastigotes inoculated by the insect vector, it is a consensus that this encounter is not the most likely, given that neutrophils, which can also phagocytose promastigotes, are the first cells to arrive at the infection site. Indeed, intravital microscopy experiments using natural infection, through the bite of infected sand flies, showed that neutrophils appear as early as 30 seconds following host exposure to sand flies and are the first cells to internalize the parasites (20). Neutrophils are attracted by the mechanical injury caused by the bite, by the sand fly’s saliva, and by the release of neutrophil chemotactic factor and IL-8 by local cells (21, 22). It has also been demonstrated that *L. major* can modulate the viability of infected neutrophils, which can delay or accelerate their death through apoptosis (23–26). The modulation of neutrophil viability plays a crucial role in the parasite survival until it reaches its final targets, the macrophages, which are attracted to the infection site by the release of chemotactic factors, such as CCL2, and remove apoptotic bodies (27, 28). This process is accompanied by the inhibition of IL-12 secretion and the elevated secretion of IL-10 and TGF-β, which together create an anti-inflammatory environment that favors parasite survival. The initial infection of neutrophils, followed by phagocytosis of infected apoptotic cells by macrophages, is known as the “Trojan horse” mechanism (29). Until very recently, it was believed that neutrophils would not allow the parasite to transform into amastigotes or replicate. However, recent studies have shown that *L. mexicana* can replicate within neutrophils, demonstrating that this cell type can also serve as a niche for parasite proliferation (30). The early infection events regarding neutrophil recruitment, the molecular events triggered inside neutrophils upon infection, and their interaction with macrophages and DC have been recently reviewed (31).

A recent study using intravital microscopy has shown increased apoptosis in macrophages infected by *L. major*, with the infection of new cells occurring without the appearance of parasites in the extracellular milieu. Amastigote forms were transferred to uninfected macrophages along with cellular material from the originally infected macrophage (32). The same study demonstrated that high pathogen proliferation increases infected cell death, suggesting that this is a parasite-driven mechanism to promote its own dissemination. The induction of macrophage apoptosis or necrosis by *L. amazonensis* or *L. guyanensis*, respectively, has already been shown *in vitro* by our group (33). These findings reinforce the Trojan horse mechanism as an important route for *Leishmania* spp. dissemination in mammalian hosts even during the chronic phase of the disease, from macrophage to macrophage. Much is discussed about the ability of *Leishmania* spp. to infect and establish themselves in macrophages, which is credited to the parasite’s extraordinary capacity to inhibit or counteract the effector functions of the phagocyte, notably the production of reactive oxygen species and nitric oxide (NO), molecules highly toxic to the parasite (34–39).

## DENDRITIC CELLS

DCs are also host cells for *Leishmania* spp. and potent APCs. Located in the epidermis, Langerhans cells (LC) and immature DCs are available at the time and site of inoculation of metacyclic promastigotes by the insect vector. However, as evaluated by *in vitro* experiments, only amastigotes efficiently enter LC-like DC, inducing upregulation of major histocompatibility complex (MHC) class I and II and of costimulatory molecules (CD40, CD54, CD80, and CD86), essential for T cell activation (40, 41), probably by the stimulation of TLR2 and/or TLR4 (41). The infection of DC by amastigotes, and not by promastigotes, is corroborated *in vivo* by the fact that 1 day after infection, an extremely small proportion of DCs are infected with parasites that are mostly inside neutrophils and a small number of macrophages. From 2 to 7 days after infection, this order is completely inverted for macrophage/neutrophil, with parasites being found primarily in the macrophage/monocyte population. By day 6, the number of infected dermal DCs and/or Langerhans cells also increases, albeit marginally (20, 26). After being infected, DCs migrate to lymph nodes, where they present parasite peptides to T lymphocytes (42). Importantly, depending on the species of *Leishmania*, infection of DC induces the release of functional interleukin (IL)−12, decisive for the development of the Th1 response, with the production of IFN-γ, which ultimately activates macrophages to produce NO, toxic for the parasites (38, 40, 43–46). It has also been reported that TLR9 is required for the induction of IL-12 in DCs by *L. major* intact parasites or DNA and for early IFN-γ expression and contributes to healing of cutaneous lesions (47, 48). Moreover, recruitment of neutrophils to the infection foci, stimulated by the release of chemoattractants by DC, is dependent on TLR9, which is upregulated during *L. infantum* infection. This neutrophil recruitment decreases the parasite number in the spleen and liver, contributing to host resistance (49).

None of these activation pathways are triggered in macrophages solely by the internalization of promastigotes or amastigotes, suggesting that these cells may not contribute significantly to the initiation of a protective immune response (40, 50). Together, these findings suggest a particular sequence of parasite uptake, marked by a delay in DC infection and migration. This delay may provide the parasite with an opportunity to exploit the host as macrophage NO production depends on activation by IFN-γ produced by Th1 cells activated through DC-derived IL-12. Several other studies and reviews highlight the role of DC and macrophages in orchestrating the immune response to *Leishmania* infection, indicating that the outcome of this parasitic infection in resistant and susceptible mice may be related to functional differences in these cells during the differentiation process of CD4 +T helper cells into effector cells (38, 51–56).

## NON-PHAGOCYTIC CELLS

For a long time, non-phagocytic cells were overlooked in leishmaniasis research, despite extensive evidence showing that the infective competence of *Leishmania* spp. extends beyond professional phagocytes, as reported both *in vivo* (57–75) and *in vitro* (60, 66, 67, 74, 76–106). Several studies describe the occurrence of inflammatory myopathy, with the presence of amastigotes in ocular, skeletal, masticatory, cardiac, and pulmonary muscle tissues of infected dogs (61, 62, 67). The presence of myopathy related to leishmaniasis has also been observed in the skeletal muscle of hamsters infected with *L. infantum* and mice infected with *L. amazonensis* (63, 107). *In vitro* studies report the internalization and replication of several species of *Leishmania* in muscle cells of different origins. The presence of amastigotes within muscle fibers was confirmed in an *in vivo* study comparing *L. amazonensis* infection in muscle cells of BALB/c and C3H.HeN mice, which exhibit different susceptibilities to leishmaniasis. While BALB/c mice, extremely susceptible to infection, showed an intense inflammatory infiltrate among myofibers infected by amastigotes, followed by muscle destruction, C3H.HeN mice, a resistant strain, displayed only mild inflammation without much damage to muscle fibers and no internalized amastigotes (63), showing a clear correlation between myofiber infection and damage with the severity of the disease. Other studies have demonstrated the establishment of infection within human placental epithelial cells, kidney cells, and mast cells, among others (108). Fibroblasts are particularly reported as being infected with *Leishmania* spp. It has been demonstrated that *L. amazonensis*, *L. mexicana*, *L. braziliensis,* and *L. major* can invade and differentiate into amastigotes within these cells (90, 103, 104, 106). An important recent discovery from our group was that *L. amazonensis* promastigotes are capable of invading fibroblasts independently of the activity of the host cell cytoskeleton—a non-phagocytic route of entry—by subverting the lysosome-dependent plasma membrane repair mechanism triggered upon contact with the parasite (106). Figure 2C shows flagellated promastigotes shortly after cell invasion in fibroblasts, while Fig. 2D shows the round-shaped amastigotes; in both cases, parasites are surrounded by lysosomal markers characterizing the maturation of the PV (as previously published by our group) (106). Like other non-phagocytic cells, fibroblasts have a limited capacity to eliminate the parasite, allowing it to survive for extended periods (66, 85, 104). Fibroblasts are important cellular components in inflammatory reactions. When activated by molecules released when tissues are damaged or derived from invading microorganisms, they can produce chemokines that attract neutrophils and macrophages (109). In fact, these cells display multiple immune features, including antigen presentation (109). Since fibroblasts are long-lived cells, it has also been proposed that they could serve as a parasite reservoir, allowing parasite latency (66), as extensively reviewed recently (110).

In CL, amastigote-induced lesions can spontaneously heal over time due to the slow development of IFN-γ-mediated responses, which activate macrophages to eliminate the parasite through the production of their characteristic antimicrobial effectors (111, 112). However, infection reactivation after lesion healing or even after drug treatment is frequent, leading to relapses (6, 113, 114). This aligns with the fact that non-phagocytic cells, which typically do not produce antimicrobial effectors, may serve as a parasite hideout during the chronic phase of the disease. In addition to serving as reservoir niches for the parasite, from which macrophages can become infected in a “Trojan horse” manner, non-phagocytic cells may also shield parasites from therapeutic drugs such as meglumine antimoniate or amphotericin-B, which primarily target infected macrophages. The mechanism of action of meglumine antimoniate is not yet fully understood, but it is thought to be both direct, by inhibiting the parasite’s glycolytic and oxidative pathways (115), and indirect, by inducing macrophages to produce anti-microbial effectors that kill the intracellular parasites (116, 117). On the other hand, lipid formulations of amphotericin B, which are more effective and less toxic, are based on the concept of targeted drug delivery to macrophages in the liver, spleen, and bone marrow, organs affected in VL (118). Thus, it is possible that parasites living in non-phagocytic cells are not reached by these two reference drugs.

Although, for decades, non-phagocytic cells have been reported to be infected *in vivo* in mammalian hosts, including humans, the passage of *Leishmania* spp. through these cells as well as their role in the parasite’s life cycle and disease progression remains a neglected topic that requires further investigation.

## INFECTION AMPLIFICATION—A STILL UNSOLVED ENIGMA

A still not understood aspect of the *Leishmania* spp. life cycle is the amplification of the infection after the initial cell invasion by promastigotes in mammals. Once internalized, the parasites travel through the endosomal pathway, establishing themselves in PVs, where they replicate. From a mechanistic point of view, little is known about how new macrophages become infected following a sand fly bite, after promastigotes have already been internalized. Since free promastigotes are no longer available, intracellular amastigotes are the only infective form responsible for amplifying the infection within the mammalian host. Indeed, it has been exhaustively demonstrated that these forms are capable of directly infecting macrophages (40, 119, 120) and other cell types (66, 78–81, 84, 86–88, 101, 103, 119).

**Fig 4:**
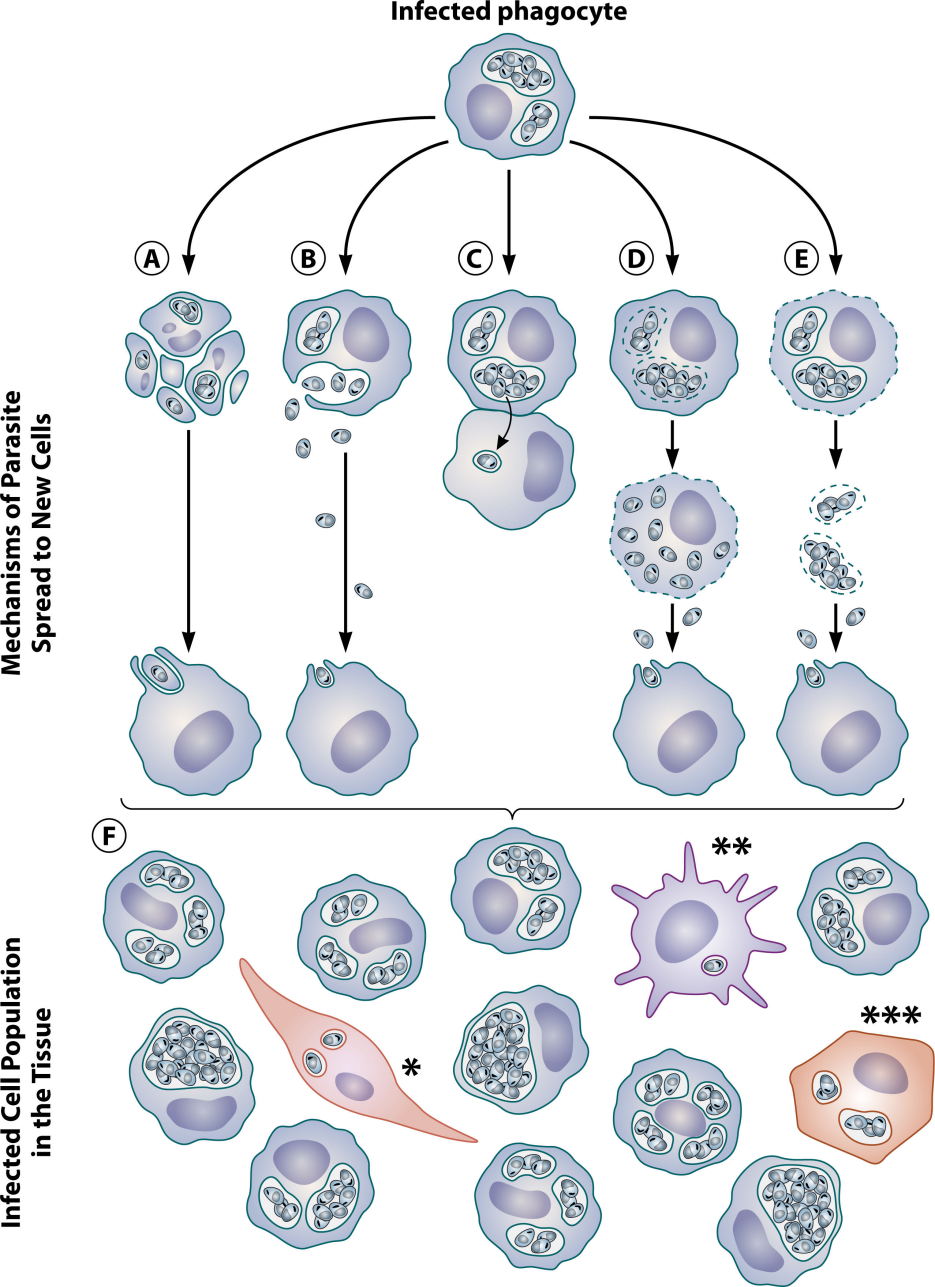
Proposed mechanisms leading to infection amplification in mammalian hosts infected by *Leishmania* spp. Upon cell invasion, *Leishmania* spp. reside in intracellular vacuoles as amastigote forms, the sole evolutionary form capable of reproducing and propagating in mammals. From the beginning of infection, the number of parasites as well as the number of infected cells increase, leading to infection amplification by still elusive mechanisms. Some possible mechanisms that have been proposed include (A) apoptosis-driven amplification—infected cells undergo apoptotic death, and their fragments containing intracellular amastigotes are phagocytosed by new macrophages. (B) Exocytosis-like release of amastigotes—amastigotes are released in the extracellular milieu after fusion of the PV with the host cell plasma membrane in an exocytosis-like manner, and the released amastigotes can infect surrounding phagocytic or non-phagocytic cells. (C) Cell-to-cell transfer—parasites can be transferred from an infected cell to a new one after close interaction. (D and E) Necrosis-like death—(D) PV membrane disrupts, releasing parasites into host cell cytosol, followed by plasma membrane rupture, releasing amastigotes in the extracellular milieu, which can infect surrounding phagocytic or non-phagocytic cells. (E) host cell plasma membrane is disrupted after the parasitized cell death, infected vacuoles are released, and can be captured by new phagocytes. In the sequence, parasites could provoke the degradation of the PV membranes, leading to the delivery of free amastigotes, which could infect surrounding cells or be captured by the phagocytes. (F) Macrophages are the majority of infected cells found in lesions, but other cell types can also be found harboring amastigotes in infected tissues, such as fibroblasts*, dendritic cells**, and epithelial cells ***.

Several possible mechanisms of amastigote transfer to uninfected macrophages are depicted in Fig. 4, of which the most currently accepted, by *in vitro* and *in vivo* demonstrations, are i) the uptake of infected apoptotic cells or apoptotic bodies by uninfected macrophages (Fig. 4A) (20, 32); ii) the release of amastigotes by an exocytosis-like mechanism, resulting in the release of amastigotes in the extracellular milieu (Fig. 4B) (108, 121); iii) the cell-to-cell transfer of amastigotes wrapped in PV membranes (Fig. 4C) (32, 77). The cell-to-cell transfer and the transit to new host cells within apoptotic bodies offer advantages to the parasite as these processes: i) occur silently (122, 123), preventing the activation of macrophage’s pathogen-killing mechanisms (124), thereby facilitating both survival and dissemination of the pathogen; and ii) reduce the risks of amastigote elimination in the extracellular environment by immune mechanisms, such as complement system activation (125), which could otherwise clear the pathogen from the host.

One mechanism usually mentioned in textbooks and represented in *Leishmania* spp. life cycle diagrams is the release of amastigotes by the rupture of infected macrophages and the uptake of free parasites by uninfected macrophages, which could occur by the rupture of phagolysosomes, followed by the rupture of the infected cell (Fig. 4D) or the inverse order (Fig. 4E). The rupture caused by a heavy amastigote load has been questioned in the past by the description by our group of a pore-forming cytolysin in *L. amazonensis* and *L. guyanensis*, called leishporin, active at acidic pH and 37°C. Leishporin, whose nature is still unclear, was proposed to be the cause of a possible phagolysosome and plasma membrane disruption (126, 127). Also in this sense, we have demonstrated that macrophages die from necrosis induced by *L. guyanensis* (33). However, vacuoles containing amastigotes are not commonly found in the extracellular environment, either *in vitro* or *in vivo*. Similarly, amastigotes are not typically found freely in the host cell cytoplasm, a stage that would precede its delivery from cells. Likewise, extracellular free amastigotes are not consistently found in the extracellular milieu. However, some studies have reported them (33, 121). In any case, the hypothesis of amplification through the infection of free amastigotes or vacuoles containing amastigotes still requires *in vivo* experimental validation.

## VISCERALIZATION AND PARASITE DISSEMINATION—CENTRAL BUT STILL ELUSIVE PROCESSES

As previously mentioned, the clinical manifestations of the different types of leishmaniasis include localized or disseminated skin lesions, the mucocutaneous form of the disease, and visceral leishmaniasis (128, 129). The underlying mechanism leading to parasite dissemination, whether from the inoculation site throughout the dermis and mucous membranes in cases of multiple lesions or DL, or to the liver, spleen, or bone marrow, in VL, remains elusive. Since parasites in the bloodstream or free amastigotes in tissues are not hallmarks of infections by *Leishmania* spp., it is reasonable to suggest that parasitized cells with the ability to migrate throughout the skin or from the skin to the organs are carriers of amastigotes. An effective immune response depends on the ability of leukocytes to migrate through tissues to sites of infection and inflammation. Additionally, the dissemination and homing of infected cells containing *Leishmania* spp. antigens are crucial for parasite survival in the host and for disease establishment. Like other pathogens, once internalized, *Leishmania* spp. can modulate the migration of host cells. Despite being the main host cell for *Leishmania* spp., the adhesion and migration of macrophages during infection are poorly understood. Some studies have shown a reduction in adhesion and migration of macrophages following infection with different *Leishmania* spp. (130–136). A recent study demonstrated a differential modulation of macrophage migration after infection depending on the species involved (137).

DCs are also known to have their migration modulated by *Leishmania* spp. As previously mentioned, studies have shown that Langerhans cells phagocytose *L. major in vivo* and migrate to the draining lymph nodes (131, 138). A study using a murine model has shown a reduction in the migratory population of DCs infected with *L. amazonensis* compared to uninfected cells, indicating that the parasite can alter the migratory pattern of these cells *in vivo* (139). It is possible that different species or hosts may favor different patterns of migration of infected cells, resulting in distinct clinical manifestations, from localized forms to diffuse ones or even visceral infections. For instance, when infected with *L. infantum*, DCs greatly increase their migratory ability, as compared to uninfected cells (140). On the other hand, the migration of *L. braziliensis*-infected DCs is considerably reduced, while *L. amazonensis*-infected DC exhibit a transient and modest increase. The migratory ability of infected DC is strongly associated with the expression of i) p-FAK and p-paxillin, both involved in adhesion complex formation; ii) RhoA, Rac1, and Cdc42, which are key regulators of actin polymerization; and iii) CCR7, which is crucial for DC migration to draining lymph nodes (141). In fact, it has been shown that CCR7 plays a role in the development of VL (142). These findings indicate that the increased migration observed in DCs infected with parasites isolated from DL and VL, but not from CL, may play a role in parasite dissemination within the vertebrate host (140). Furthermore, a recent study using an MCL model showed that lymphatic vessels can act as effective pathways for infected cells to leave the primary site and spread to distant organs when infection is accompanied by inflammation and exacerbated by the presence of the viral endosymbiont (LRV1) (143). However, the precise role of different host cells in the parasite’s dissemination within the vertebrate host, as well as the molecular mechanisms involved, still requires further elucidation.

It is important to highlight that parasite dissemination across skin or organs is not a trivial process as it is driven by multiple factors including parasitic virulence as well as the host’s genetics, age, and nutritional and immunological status. Moreover, the inflammatory immune response must be tightly regulated to prevent tissue damage and/or avoid inducing a self-regulatory feedback loop that impairs the elimination of the parasite (144, 145). *Leishmania* spp. has evolved not only by controlling the host’s immune system but also by exploiting it.

## INSIDE THE SAND FLY VECTOR

### Resuming the multiplication stage to boost the vector’s infectivity

The life cycle of *Leishmania* spp. proceeds when sand flies, during a bloodmeal on an infected mammal, become infected by ingesting cells containing amastigotes (Fig. 3F and G), which will re-differentiate into multiplicative procyclic promastigotes, located at the midgut of the insect (Fig. 3G and H). They differentiate into nectomonad promastigotes, a non-dividing migratory stage moving from the sand fly posterior to the anterior midgut. There, they differentiate into leptomonad forms, which resume replication (Fig. 3H and I). Leptomonad promastigotes then undergo the so-called metacyclogenesis (Fig. 3I and K), a process involving different stages of biochemical and morphological modifications that culminate in the emergence of nonreplicative metacyclic promastigote forms, highly infective to the vertebrate host. Leptomonad promastigotes secrete a gel that forms a physical obstruction in the gut, forcing the sand fly to regurgitate metacyclic promastigotes into the host dermis during blood meal, which plays a key role in the transmission of the parasite to mammals (146). In the recent past, it was believed that the transformation of the leptomonad stage into highly infective metacyclic forms was a terminal differentiation process. Interestingly, however, it has been demonstrated for *L. major* and *L. infantum* that the ingestion of a second blood meal by infected female sand flies in non-infected hosts triggers a dedifferentiation of metacyclic promastigotes into a leptomonad-like stage, in a kind of “reverse metacyclogenesis” (146). This allows parasites to resume cell division and rapidly redifferentiate into metacyclic promastigotes, dramatically increasing parasitic load, leading to superinfective vectors (146) (Fig. 3K and I, asterisk). These recent findings highlight the central role of the blood as a critical element in the parasite cycle, directly impacting transmission, propagation, and infectivity in *Leishmania* spp.

### Genetic exchange between parasites

For several decades, *Leishmania* spp. were thought to reproduce predominantly through a clonal mode, with recombination considered an exceptionally rare event (147). However, advances in laboratory techniques and sophisticated statistical analyses have challenged this clonal-only hypothesis, revealing a more nuanced understanding of the reproductive biology of *Leishmania*. A genetic study examining 12 loci across 125 strains of *L. braziliensis* infecting humans demonstrated high levels of homozygosity, indicative of mating between closely related lineages (148). Supporting this, experiments using transgenic strains resistant to distinct selective drugs have shown genetic exchange in *L. major* within sand fly vectors. Though rare, these events could explain the hybrid genotypes observed in field isolates of various *Leishmania* species (149). This evidence suggests that *Leishmania* may alternate between different reproductive modes: clonal reproduction within both vertebrate and insect hosts and inbreeding within the vector.

Recent breakthroughs have provided additional evidence for meiosis-like recombination in *Leishmania* spp. A study employing whole-genome sequencing of experimental hybrids highlighted recombination patterns consistent with meiosis (150). Furthermore, a unique population of hybridization-competent promastigotes expressing orthologs of meiotic genes was identified after gamma radiation exposure, suggesting meiosis-like mechanisms in *Leishmania* spp (151). An exciting discovery highlights the involvement of natural IgM antibodies (IgMn) from uninfected animals in the mating process. These antibodies bind to the surface of promastigotes, inducing the formation of spherical aggregates that facilitate genetic exchange, parasite fusion, and hybrid formation, increasing hybridization events by 12-fold (152).

Progress in unraveling the reproductive biology of *Leishmania* spp. has been hampered by several challenges. These include the limited availability of vector sand flies and genetically modified parental strains necessary for generating hybrids, as well as the facultative nature of genetic exchange, which results in a low frequency of hybrid formation under experimental conditions. However, a significant recent milestone has been the development of methods to generate stable hybrids entirely *in vitro*. By subjecting cultured promastigotes to DNA stress, researchers have significantly enhanced the frequency of hybridization, facilitating genetic exchange both within and between different *Leishmania* species (151, 153).

Another issue related to genetic exchange that warrants attention is the inheritance of extrachromosomal circular DNA encoding drug resistance, which holds significant interest and potential applicability. Recent studies have demonstrated that *Leishmania* spp. can exchange drug resistance genes through extracellular vesicles transferred between parasites (154), raising the possibility that a similar pathway, involving microvesicles, could mediate a broader exchange of genetic material, contributing to the complexity of the scenario.

## CONCLUDING REMARKS

The various forms of leishmaniases are challenging diseases to control, with their treatment and prevention remaining significant public health concerns across multiple countries on different continents. Despite decades of studies by various groups, many aspects of the basic biology of *Leishmania* spp. remain poorly understood. In this review, we sought to bring to fore the most recent findings that address some of these knowledge gaps. Notably, we have provided an updated representation of the life cycle of *Leishmania* spp. (Fig. 3), which we believe has remained largely unchanged since its initial description, despite the long-standing evidence for certain steps and the lack of validation for others.

As discussed in this review, definitive answers to seminal questions, such as the following, remain elusive: i) What is the entire spectrum of relevant host cells for Leishmania, and what roles do they play in the life cycle of the pathogen and the pathogenesis of the different forms of leishmaniasis? ii) Which routes and mechanisms does the parasite use for migration from the site of inoculation through the skin and further on to internal organs? iii) Are there additional mechanisms beyond these already proposed (Fig. 4) that enable amastigotes to invade new cells and amplify the infection? Understanding the basic biology of different species of *Leishmania* and their vectors, as well as the intricate evolutionary relationship between them, is crucial to comprehending the infectious process and to developing strategies for prophylaxis, treatment, and control of this global parasitic threat.
